# The Frequency of Rotavirus Gastroenteritis in Children from West of Iran and Genotyping of Rotavirus Isolates: A Suggestion for Further Changes in Childhood Immunization Program

**DOI:** 10.34172/jrhs.2024.156

**Published:** 2024-07-31

**Authors:** Parinaz Sedighi, Manoochehr Karami, Mehta Razzaghi, Maryam Emamjamaat, Abdollah Karimi, Roxana Mansour Ghanaiee, Masoud Alebouyeh, Iraj Sedighi

**Affiliations:** ^1^Student Research Committee, Hamadan University of Medical Sciences, Hamadan, Iran; ^2^Universal Scientific Education and Research Network (USERN), Tehran, Iran; ^3^Environmental and Occupational Hazards Control Research Center, Research Institute for Health Sciences and Environment, Shahid Beheshti University of Medical Sciences, Tehran, Iran; ^4^Department of Epidemiology, School of Public Health and Safety, Shahid Beheshti University of Medical Sciences, Tehran, Iran; ^5^Clinical Research Development Unit of Besat Hospital, Hamadan University of Medical Sciences, Hamadan, Iran; ^6^Department of Pediatrics, School of Medicine, Hamadan University of Medical Sciences, Hamadan, Iran; ^7^Pediatric Infections Research Center, Research Institute for Children’s Health, Shahid Beheshti University of Medical Sciences, Tehran, Iran

**Keywords:** Rotavirus, Rotavirus vaccines, Gastroenteritis, Vaccination, Immunization programs

## Abstract

**Background:** Rotavirus is the most common cause of gastroenteritis among children. Currently, four oral live-attenuated vaccines are available to prevent rotavirus infection. The World Health Organization (WHO) has recommended including rotavirus vaccination in national immunization programs; however, it has not been introduced to the Iranian national immunization program. The study aimed to assess the frequency of rotavirus gastroenteritis in the west of Iran and investigate the necessity of rotavirus vaccination.

**Study Design:** A case series study.

**Methods:** In this case series study, 284 cases under six years of age who presented with acute gastroenteritis from March 2021 to 2022 to a referral hospital in the west of Iran were evaluated. Data on baseline characteristics, clinical manifestations, results of stool test, ELISA for rotavirus detection, and polymerase chain reaction (PCR) test for genotyping of rotavirus-positive samples were recorded.

**Results:** Results showed that the prevalence of rotavirus infection was 36.6%. The highest frequency was observed among children aged 6-12 months and during the autumn. According to the PCR results, G1P[8], G9P[8], G9P[4], and G1P [4] were the dominant genotypes, and 33.75% of samples were infected with multiple rotavirus genotypes.

**Conclusion:** The study highlights the considerable prevalence of rotavirus infection among cases of acute gastroenteritis in children under six years of age who were referred to a referral hospital in the west of Iran and the high diversity of rotavirus genotypes in the targeted community. Consequently, physicians and health policymakers should prioritize strategies for the prevention and control of this infection, particularly by considering the rotavirus vaccine as a priority for the Iranian national immunization program.

## Background

 Diarrhea is one of the leading causes of morbidity and mortality among children. Rotavirus is the primary cause of gastroenteritis in children under five years of age.^1,2^ Before the introduction of the rotavirus vaccine in 2006, almost all children contracted the disease between the ages of 3 and 5. According to the World Health Organization (WHO), rotavirus was responsible for more than 2 million hospitalizations due to gastroenteritis and over 500 000 childhood deaths in 2000. Statistics from 2016 indicate that there were 287 million episodes of diarrhea and 128­500 deaths due to rotavirus infection in children under five worldwide.^3,4^ Today, despite the significant reduction in severe gastroenteritis rates in children following the implementation of the rotavirus vaccine, rotavirus remains the most common cause of gastroenteritis in children under five years old.^1,3^

 Rotaviruses are triple-layered particles containing 11 segments of double-stranded ribonucleic acid (RNA). The outer capsid layer of rotavirus contains two main antigens called viral protein-4 and viral protein-7 (VP4 and VP7). The VP4 determines the P-type and the VP7 determines the G-type of rotaviruses. Before vaccination, era G1P[8], G2P[4], G3P[8], G4P[8], and G9P[8] were the dominant strains worldwide but after vaccination, the pattern of common strains has varied in several countries across the world.^1-3^

 The symptoms of rotavirus gastroenteritis include fever, vomiting, and non-bloody watery diarrhea. Severe cases present with significant dehydration, seizure, and even death in some cases.^5^ Diagnosis is based on the detection of viruses in patients with clinical manifestations of gastroenteritis. Enzyme-linked immunosorbent assay (ELISA) is the best and the most available method for detecting rotavirus with a sensitivity and specificity of more than 90%.^6^

 Currently, there are four oral live-attenuated vaccines available internationally for the prevention of rotavirus gastroenteritis including RotaTeq (Merck & Co. Inc., Whitehouse Station, NJ, USA), Rotarix (GlaxoSmithKline Biologicals, Rixensart, Belgium), Rotavac (Bharat Biotech International Ltd, India), and ROTASIIL (Serum Institute of India, India). RotaTeq is a pentavalent human-bovine vaccine (G6P[5]) that is administered in a 3-dose schedule. Rotarix is a monovalent human vaccine (G1P[8]) that should be administered in a 2-dose schedule. ROTASIIL (pentavalent, human G1, G2, G3, G4, G9 strains, and bovine G6P[5] strain) and Rotavac (monovalent, G9P [11] strain) are human-bovine vaccines that should be administered in a 3-dose schedule.^3^ Several studies have shown that vaccine implementation had 90%-95% protection against severe rotavirus gastroenteritis in countries with low mortality rates. Moreover, the prevalence rate among countries with high mortality rates was 44%-70%.^7^

 Until 2022, 114 countries have introduced rotavirus vaccines into their national vaccination program; however, the vaccine has not been included in the Iranian national immunization program.^8,9^ Regarding WHO’s recommendation to include rotavirus vaccine in national immunization programs of all countries, we aimed to perform this study to assess the frequency of rotavirus gastroenteritis among Iranian children and to assess the necessity of vaccination in Iran.^3^

## Materials and Methods

 In this descriptive (case series) study, patients younger than six years old who presented with acute gastroenteritis to Besat Hospital (Hamadan, West of Iran) from March 2021 to March 2022 were included after receiving informed consent. The written informed consent was obtained from patients’ parent or legal guardian by physicians of the team after providing information about the process and aims of the study. Cases of diarrhea lasting more than 14 days, diarrhea due to chronic diseases like ulcerative colitis and Crohn’s disease, antibiotic-associated diarrhea, and bloody diarrhea were excluded from the study. Clinical and demographic data including gender, age, type of nutrition (breastfed, formula, mixed, and varied nutrition), type of admission (inpatient or outpatient), duration of hospital stay, presence of fever and vomiting, number of diarrhea and vomiting episodes, presence of dehydration, type of fluid therapy, complications including seizure, and need for pediatric intensive care unit (PICU) admission were entered into a checklist for each patient. Besides, results of laboratory tests including stool tests (microscopic evaluation, chemical tests, white blood cell (WBC) and red blood cell (RBC) counts, and microbial analysis or culture), ELISA for rotavirus detection, and polymerase chain reaction (PCR) test for genotyping of rotavirus-positive samples were added to the checklists in the next steps.

 A stool sample was obtained from each patient and a stool test was performed in the first step. In the second step, ProSpecT^TM^ Rotavirus Microplate Assay kit (Oxoid, Ltd. UK) was used for the ELISA test. ProSpecT^TM^ Rotavirus Microplate Assay is an immunoassay method for the detection of group A rotaviruses in fecal specimens. The test uses polyclonal antibodies in a solid phase to detect group-specific antigens including major inner capsid protein (VP6). For performing the ELISA test, a 10% dilution of fecal specimen was provided by adding 0.1 g of solid fecal sample or 100 μL of liquid fecal to 1 mL of diluent provided with the kit. Then, 100 μL of the suspension was added to separate microwells. Additionally, 100 μL of negative and 100 μL of positive control were added to two microwells for each batch of testing. After the addition of solutions and controls, 100 μL of the conjugate (provided with the kit, including rabbit polyclonal antibody conjugated to horseradish peroxidase) was added to the microwells, and microwells were incubated for 60 minutes at 20-30 ℃. After binding of antigens and antibodies during the incubation period, fluid was aspirated and microwells were washed six times (wash buffer was available in the kit) to remove unbound antibodies. Then, 100 μL of the chromatogen liquid was added to each microwell and 100 μL of stop solution was added after 10 minutes. Finally, the absorbance of each microwell was read by an ELISA reader spectrophotometer at 450 nm. To calculate the cut-off value, 0.2 absorbance units were added to the negative control value. Samples with absorbance values exceeding the cut-off were classified as positive, while those below were considered negative. Values falling within 0.01 units of the cut-off were deemed equivocal.

 In the third step, PCR tests were done on samples that were considered positive or equivocal according to the ELISA test. Diluted fecal specimens in phosphate-buffered saline (PBS) were used for virus detection. RNA extraction from the suspended fecal samples was done using a High Pure Viral RNA extraction kit (Roche, Germany) according to the manufacturer’s protocol. The RNA extracts were stored at -70 ℃ until use for reverse transcription PCR (RT-PCR). Complementary Deoxyribonucleic Acid (cDNA) synthesis and nested multiplex PCR were used for G and P genotyping of VP7 and VP4 genes using type-specific primers (SinaClon, Iran) according to the standard rotavirus type A detection and characterization method of WHO guideline.^10^ The extracted RNA was denatured by heating at 94 °C for 5 minutes and then chilled at 4 ℃ to be used for cDNA synthesis. The cDNA was synthesized with AddScript cDNA Synthesis Kit (Cat. No. 22701, Addbio, Korea), using a single cycle in a final volume of 13.2 μL as follows: 10 minutes at 25 °C, 30 minutes at 42 °C, and 5 minutes at 80°C. First-round PCR reaction was used to amplify the genes with the forward (GGCTTTAAAAGAGAGAATTTCCGTCTGG) and reverse (GGTCACATCATACAATTCTAATCTAAG) primers for VP7, and forward (TGGCTTCGCTCATTTATAGACA) and reverse (ATTTCGGACCATTTATAACC) primers for VP4.^10^ The annealing temperatures for type-specific primers were as follows: for G-specific primers, 42 °C and 50 °C for the first and second run, and for P-specific primers, 52 °C and 50 °C for the first and second run, respectively. After agarose gel electrophoresis to characterize related genotypes in each sample, single PCR using specific primers (SinaClon, Iran) for each genotype was used to confirm the results in cases infected with multiple rotavirus genotypes (A, G, and P).

 Data analysis was administered using SPSS version 22.0 (IBM Corporation, Armonk, New York). Quantitative variables were reported as median, mean, and standard deviation. Qualitative variables were reported as frequency and percentage. The estimates on proportions were reported by percentage for point estimate and 95% confidence intervals around the point estimate. Rotavirus-positive and rotavirus-negative groups were compared using chi-square test and *t* test for qualitative and quantitative variables, respectively. A *P* value of less than 0.05 was considered statistically significant.

## Results

 A total of 284 children under six years old, presented with acute gastroenteritis, were included in the study. Based on the results, 172 cases (60.6%) were boys. The mean age of participants was 21.48 ( ± 22.5) months. The median and mode of age were 13.5 and 6 months, respectively. Most of the patients (45.4%) were 12 months old or younger and the number of patients decreased with age. Additionally, 7.4% of cases were detected during the last month of winter in 2021, and 16.5%, 28.2%, 24.3%, and 23.6% of cases were detected during spring, summer, autumn, and winter of 2022, respectively.

 In terms of clinical manifestations, 87.3% and 68.7% of the cases had fever and vomiting on admission, respectively. Besides, 37% of cases presented with dehydration. Moreover, one patient (0.4%) presented in a dehydration shock state. Finally, for prevention or treatment of dehydration, 25.4% of patients received oral rehydration therapy (ORT), 56.3% received intravenous (IV) hydration, and 14.1% received both ORT and IV hydration. Of 284 cases, 22 cases developed gastroenteritis-related complications, 19 of whom presented with febrile convulsion (FC).

 According to laboratory findings, exudative diarrhea, defined as more than 5 WBC/hpf (WBCs per high-power field of the microscope), was detected in 17.3% of cases.

**Figure 1:**
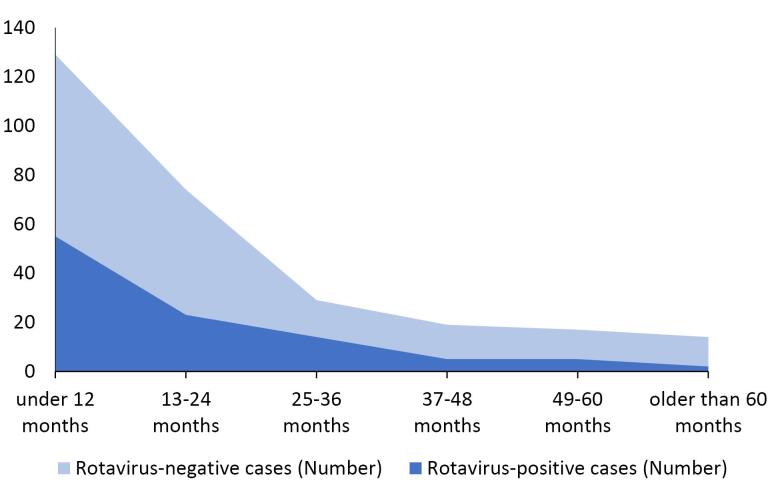
Age distribution of rotavirus gastroenteritis

 Based on the results of the ELISA test, rotavirus was positive in 104 cases (36.6% with 95% confidence intervals: 31.0-42.5), negative in 171 cases (60.2%), and equivocal in 9 cases (3.2%). The prevalence of rotavirus infection among girls and boys was equal (36.6%). Most rotavirus-positive cases (52.9%) were children less than or equal to 12 months old. Moreover, 15.8% were under six months and 37.5% were 6-12 months. The prevalence of rotavirus infection decreased with age (Table 1), although there was no significant difference among the six age groups regarding the prevalence of rotavirus infection. After dividing patients into two age groups (older and younger than three years) and deleting the equivocal results, there was a significant difference in the prevalence of rotavirus infection between these two groups (*P* = 0.049) (Table 2). The decreasing trend of the rotavirus infection rate with increasing age is shown in Figure 1.

**Table 1:** Rotavirus enzyme-linked immunosorbent assay (ELISA)^a^ results according to age

**Age group (months)**	**Positive, n=104**	**Negative, n=171**	**Equivocal, n=9**	**Total, n=284**
Less than or equal to 12	55	70	4	129
13-24	23	49	2	74
25-36	14	14	1	29
37-48	5	13	1	19
49-60	5	12	0	17
More than 60	11	11	1	14
Undetermined	0	2	0	2

^a^To calculate the cut-off value, 0.2 absorbance units were added to the negative control value. Samples with absorbance values exceeding the cut-off were classified as positive, while those below were considered negative. Values falling within 0.01 units of the cut-off were deemed equivocal.

**Table 2:** Rotavirus enzyme-linked immunosorbent assay (ELISA) results for two age groups (*P* = 0.049)

**Age groups (y)**	**Positive**		**Negative **		**Total**	
	**Number**	**Percentage**	**Number**	**Percentage**	**Number**	**Percentage**
< 3	93	88.6	132	78.6	225	82.4
≥ 3	12	21.4	36	11.4	48	17.6
Total	105	100	168	100	273	100

**Figure 2:**
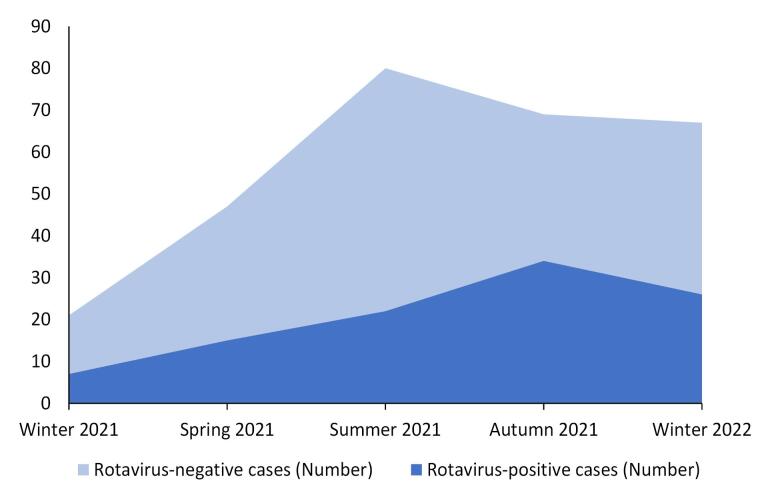
Seasonality of Rotavirus gastroenteritis

 Most cases of gastroenteritis among children were detected during summer (28.2%), although the highest prevalence of rotavirus gastroenteritis was reported during autumn (49.3%). The total number of gastroenteritis and the number of rotavirus-positive cases are shown in Figure 2. After omitting the equivocal ELISA results, 31.1% of gastroenteritis cases during warm seasons (spring and summer) and 43.8% of gastroenteritis cases during cold seasons (autumn and winter) were rotavirus-positive, respectively. There was a significant difference in the prevalence of rotavirus-associated gastroenteritis between warm and cold seasons (*P* = 0.034).

 In terms of clinical manifestations, 83.7% of rotavirus-positive cases and 89.5% of rotavirus-negative cases had fever. Moreover, 71.2% of rotavirus-positive cases and 67.3% of rotavirus-negative cases had vomiting. There was no significant difference between the rotavirus-positive and negative groups (*P* > 0.05).

 Comparison of parameters indicating the severity of gastroenteritis including the degree of fever, number of diarrhea and vomiting episodes, type of fluid therapy (ORT or IV hydration), the frequency of PICU admission, and the frequency of infection or dehydration-related complications showed that there was no significant difference between rotavirus-positive and rotavirus-negative groups. The prevalence of FC was 5.8% in the rotavirus-positive group and 8.2% in the rotavirus-negative group, indicating no significant difference between the two groups.

 Stool samples with positive (104 cases) or equivocal (9 cases) ELISA results were prepared for the PCR test to determine the rotavirus genotype. Some samples were excluded due to insufficient sample volume; therefore, genotyping was done for 80 samples. Based on the results of the PCR test, 53 cases (66.25%) were infected with a single rotavirus genotype. G1P[8], G9P[8], G9P[4], and G1P[4] were the most common genotypes accounting for 16.25%, 13.75%, 12.34%, and 6.25% of samples, respectively. Additionally, 27 cases (33.75%) were infected with multiple rotavirus genotypes.

## Discussion

 According to a WHO report published in 2000, rotavirus accounted for more than 2 million hospital admissions and more than 500 000 deaths among children worldwide. After the introduction of rotavirus vaccines, the rate of rotavirus-associated severe gastroenteritis has decreased significantly and vaccination prevented 20% of child deaths due to gastroenteritis; however, rotavirus is still among the main causes of gastroenteritis in children.^3,4^

 In this study, 284 patients under six years of age, who presented with acute gastroenteritis, were evaluated. Based on the results, 60.6% of cases were boys. According to ELISA test results, 36.6% were rotavirus-positive, 60.2% were rotavirus-negative, and 3.2% had equivocal results. The results of this study were consistent with the results of previous studies from Iran, studies from other countries that do not include rotavirus vaccination in their national immunization programs, and studies before vaccine introduction.^9,11,12^

 A study conducted by Aliabadi et al^11^ during 2008-2016 on more than 400 000 children less than five years old from 82 countries across the world reported that the global prevalence of rotavirus gastroenteritis was 32%. This rate varies from 23% in countries that have introduced the rotavirus vaccine to 38% in countries that do not provide rotavirus vaccination in their national immunization programs. This study showed a 39.6% decrease in rotavirus infection incidence after vaccine introduction. A review article by Shaheen^13^ on rotavirus gastroenteritis among hospitalized children under five years of age in the Eastern Mediterranean Region (EMRO) reported that the prevalence of rotavirus gastroenteritis during 2010-2016 was 42.7%. This rate varied from 22.5% to 63% among countries across the EMRO. Additionally, this study mentioned that the rate of rotavirus gastroenteritis in Iran increased from 19% to 25% from 2004 to 2014. According to our study, the prevalence of rotavirus gastroenteritis in a population representative of the west of Iran was 36.6% between 2021 and 2022. It should be noted that the study was conducted in a hospital which serves as a referral center for the western region of Iran. Another study conducted by Jalilvand et al^9^ in Iran reported that rotavirus infection accounted for 40.4% of severe gastroenteritis that required hospitalization. This study reported the rate of rotavirus gastroenteritis in Iran increased from 25% in 1986-1990 to 42.77% in 2010-2015, which confirms the upward trend in recent years. Additionally, according to a study conducted in South-Western Iran in 2009-2010, the frequency of rotavirus was 35%, which is consistent with our results.^14^ A concurrent study in Tehran, Iran, with the same methodology as ours reported a rotavirus infection prevalence of 28.5% among children with acute diarrhea. The rate was lower than our results from West of Iran.^15^

 According to our results, 60.5% of the gastroenteritis cases were boys, although the prevalence of rotavirus infection was the same among girls and boys (36.6%). According to the systematic review conducted by Shaheen,^13^ the prevalence of rotavirus infection was higher among boys, however, we did not find a difference. The mean, median, and mode of age were 21.48, 13.5, and 6 months, respectively. The results show that the data are skewed to the right and the results are consistent with previous studies that reported the highest prevalence of rotavirus infection among children aged 6-12 months.^13^

 Based on the age distribution of rotavirus infection, the highest prevalence was observed in children between 6-12 months old. This can be explained by the presence of maternal antibodies and exclusive breastfeeding until six months given that the infant does not have exposure to sources of infection from the fecal-oral route. In addition, most children acquire immunity until one year of age. According to Figure 1, the prevalence rates of gastroenteritis and rotavirus infection decrease with age, which is also reported by several studies from Iran, Morocco, and Saudi Arabia.^14,16-19^

 The results of this study showed that most cases of gastroenteritis were detected during summer; however, most cases of rotavirus-positive gastroenteritis were detected during autumn (Figure 2). Additionally, the prevalence of rotavirus infection was significantly higher during cold seasons. Similar studies reported the highest incidence of rotavirus infection during autumn.^12,13,20^ One study from Iran reported that the peak incidence occurred in summer^21^ and two studies from Poland and India reported that the peak incidence occurred in winter.^12,20^ These variations in seasonality can be a result of differences in climate (affecting the rate of transmission) in different geographical areas. One study has claimed that the Coronavirus disease-19 pandemic in 2020 affected the seasonality of rotavirus infection and the peak incidence occurred later than the previous years.^20^

 Results of this study showed that there was no significant difference in terms of the parameters indicating the severity of disease between rotavirus-positive and rotavirus-negative groups; however, previous studies reported more severe disease and a higher frequency of complications in rotavirus-associated gastroenteritis. A case-control study conducted in England during 2011-2013 declared that rotavirus causes more severe diarrhea in comparison to other microbial causes. Besides, the incidence rates of fever, need for IV hydration, need for hospitalization, and re-admission were significantly higher in the rotavirus-positive group.^22^

 The most common complication of rotavirus infection according to our study was neurological complications, especially FC, however, we did not find a clear relationship between rotavirus infection and FC. There is no consensus among the studies on this matter. Several articles have established a relationship between rotavirus gastroenteritis and FC^23-26^; however, a recent study by Karampatsas et al^22^ did not find a significant difference between rotavirus-positive and rotavirus-negative groups. Further studies are recommended to determine the exact relationship.

 According to the PCR test results, G1P[8], G9P[8], G9P[4], and G1P[4] were the most common genotypes. Moreover, the review study conducted by Shaheen, one study performed in India, and two studies conducted in Iran reported G1P[8] as the most common genotype of rotaviruses causing gastroenteritis among children.^9,12,13,27^ Our results are consistent with previous studies that showed a considerable spread of infection with multiple rotavirus genotypes.^27^

 Several studies have confirmed the role of rotavirus vaccines in preventing severe disease and lowering the prevalence of rotavirus gastroenteritis.^7^ The Global Rotavirus Surveillance Network (GRSN) reported a 40% decrease in rotavirus infections after the vaccine introduction.^11^ A full vaccination course is more effective than a single-dose vaccine; however, completing vaccination with one vaccine product or using different products did not have a significant difference.^28-30^

 Considering several epidemiologic and social parameters including the prevalence of rotavirus gastroenteritis in different countries and the costs of vaccination, WHO has recommended rotavirus vaccination be included in the national immunization programs of all countries, and the vaccine is considered cost-effective even in low-to-middle income countries. WHO has strongly recommended vaccination in areas with a high prevalence of rotavirus infection like south and south-east Asia.^3^ Despite the WHO recommendations, the rotavirus vaccine has not yet been included in the vaccination program in Iran.

 The present study has limitations as well. Although the study was conducted in a hospital that serves as a referral center for the western region of Iran, we were not able to include all children with gastroenteritis. Some cases may have been referred to other clinics. All laboratory tests were conducted with the highest possible precision, but all tests have degrees of error, and the study may contain some false positive or false negative results.

**Highlights**

- Rotavirus accounts for a significant share of acute gastroenteritis among children.
- This study detected a high diversity of rotavirus genotypes leading to gastroenteritis in children.
- Prevention and control of rotavirus infection should be a priority in the health system, particularly by considering the rotavirus vaccine.

## Conclusion

 The results of our study highlight the considerable prevalence of rotavirus infection among cases of acute gastroenteritis in children under six years old who referred to a referral hospital in the west of Iran. Additionally, our results showed a high diversity of rotavirus genotypes causing gastroenteritis in the targeted community. These results convey two main messages. First, physicians and child healthcare providers should consider rotavirus infection when encountering childhood gastroenteritis and choose supportive and therapeutic measures accordingly. Second, health policymakers should prioritize strategies for the prevention and control of this infection, particularly by considering the rotavirus vaccine as a priority for the Iranian national immunization program.

## Acknowledgements

 The authors would like to thank the staff of the microbiology laboratory at Besat Hospital (Hamadan, Iran) for their tremendous support in conducting this study.

## Authors’ Contribution

**Conceptualization:** Iraj Sedighi, Abdollah Karimi, Roxana Mansour Ghanaiee.

**Data curation:** Iraj Sedighi, Parinaz Sedighi, Roxana Mansour Ghanaiee.

**Formal analysis:** Parinaz Sedighi, Manoochehr Karami.

**Funding acquisition:** Manoochehr Karami, Iraj Sedighi, Abdollah Karimi, Roxana Mansour Ghanaiee.

**Investigation:** Parinaz Sedighi, Mehta Razzaghi, Maryam Emamjamaat, Masoud Alebouyeh.

**Methodology:** Iraj Sedighi, Manoochehr Karami, Parinaz Sedighi, Mehta Razzaghi, Masoud Alebouyeh.

**Project administration:** Parinaz Sedighi, Roxana Mansour Ghanaiee.

**Resources:** Iraj Sedighi, Roxana Mansour Ghanaiee.

**Software:** Parinaz Sedighi, Manoochehr Karami.

**Supervision:** Manoochehr Karami, Iraj Sedighi.

**Validation:** Manoochehr Karami, Iraj Sedighi.

**Visualization:** Parinaz Sedighi, Iraj Sedighi, Manoochehr Karami.

**Writing–original draft: **Parinaz Sedighi, Iraj Sedighi, Manoochehr Karami.

**Writing–review & editing:** Iraj Sedighi, Parinaz Sedighi, Manoochehr Karami.

## Competing Interests

 The authors declare that there is no conflict of interests regarding the publication of this article.

## Ethical Approval

 All investigations were done on stool samples without performing any invasive procedure. The article does not contain any personal data. The overall results were reported in the study and cases were included in the study after obtaining informed consent from the child’s legal guardian. The study was approved by the Ethics Committee of Hamadan University of Medical Sciences (IR.UMSHA.REC.1400.152).

## Funding

 This study is part of a national multi-center study, whose outcomes are underway for sharing with the scientific community. The national study is supported by the WHO according to an agreement with Pediatric Infections Research Center, Research Institute for Children’s Health, Shahid Beheshti University of Medical Sciences, Tehran, Iran, (WHO Registration 2021/1133866-0) for enhancing rotavirus surveillance system & analytic reporting in Iran. The financial support for the collection of samples was provided by Hamadan University of Medical Sciences.
